# Neuronal activity and remyelination: new insights into the molecular mechanisms and therapeutic advancements

**DOI:** 10.3389/fcell.2023.1221890

**Published:** 2023-07-26

**Authors:** Yiting Zhou, Jing Zhang

**Affiliations:** Department of Pharmacy, School of Medicine, Sir Run Run Shaw Hospital, Zhejiang University, Hangzhou, China

**Keywords:** neuronal activity, remyelination, oligodendrocytes, molecular mechanism, therapeutic advances

## Abstract

This article reviews the role of neuronal activity in myelin regeneration and the related neural signaling pathways. The article points out that neuronal activity can stimulate the formation and regeneration of myelin, significantly improve its conduction speed and neural signal processing ability, maintain axonal integrity, and support axonal nutrition. However, myelin damage is common in various clinical diseases such as multiple sclerosis, stroke, dementia, and schizophrenia. Although myelin regeneration exists in these diseases, it is often incomplete and cannot promote functional recovery. Therefore, seeking other ways to improve myelin regeneration in clinical trials in recent years is of great significance. Research has shown that controlling neuronal excitability may become a new intervention method for the clinical treatment of demyelinating diseases. The article discusses the latest research progress of neuronal activity on myelin regeneration, including direct or indirect stimulation methods, and the related neural signaling pathways, including glutamatergic, GABAergic, cholinergic, histaminergic, purinergic and voltage-gated ion channel signaling pathways, revealing that seeking treatment strategies to promote myelin regeneration through precise regulation of neuronal activity has broad prospects.

## 1 Introduction

Oligodendrocytes play a substantial role in the central nervous system by forming myelin sheaths and maintaining their structure. Myelin accelerates nerve impulse conduction, increases neural signal processing capacity, maintains axon integrity, and supports axonal nutrition. Demyelination is characterized by the loss of myelin sheaths around the axon, leading to oligodendrocyte death, which is common in clinical disorders such as multiple sclerosis (MS), chronic cerebral hypoperfusion, stroke, dementia, and schizophrenia (Fields, 2008). Though spontaneous remyelination occurs in demyelination diseases, it is generally incomplete and does not contribute to functional recovery in conditions like MS and chronic cerebral hypoperfusion (Zhou et al., 2017; Maas and Angulo, 2021; Deng et al., 2023). Several clinical trials have investigated a variety of molecules that have the potential to enhance remyelination, but their outcomes have been unsatisfactory (Lubetzki et al., 2020). Thus, effective and precise therapeutic approaches for demyelination disorders are crucial.

Multiple research groups have provided the evidences suggesting that neuronal activity could trigger increased myelin formation (Gibson et al., 2014; Mitew et al., 2018). Activation of signaling pathways between neurons and oligodendrocytes could modulate several developmental stages of oligodendrocytes, including oligodendrocyte precursor cells (OPCs) proliferation, differentiation, and maturation, as well as integrations of neural pathways (Thornton and Hughes, 2020; Deng et al., 2023). Evidence suggests that controlling neuronal excitability may be an appropriate novel intervention for clinical therapy of demyelination conditions. Neuronal activity can be stimulated directly or indirectly by various methods such as optogenetics, chemogenetics, transcranial stimulation, and sensory stimulation (Nagy et al., 2017; Maas and Angulo, 2021; Mooshekhian et al., 2022). In this review, we focus on recent advances in neuronal activity and remyelination, as well as the related mechanisms of neural signaling pathways. This outlook will help to translate experimental findings into therapeutic strategies and clinical trials aimed at enhancing remyelination and neuroprotection associated with demyelination diseases.

## 2 The role and molecular mechanisms of neuronal activity on remyelination

**FIGURE 1:**
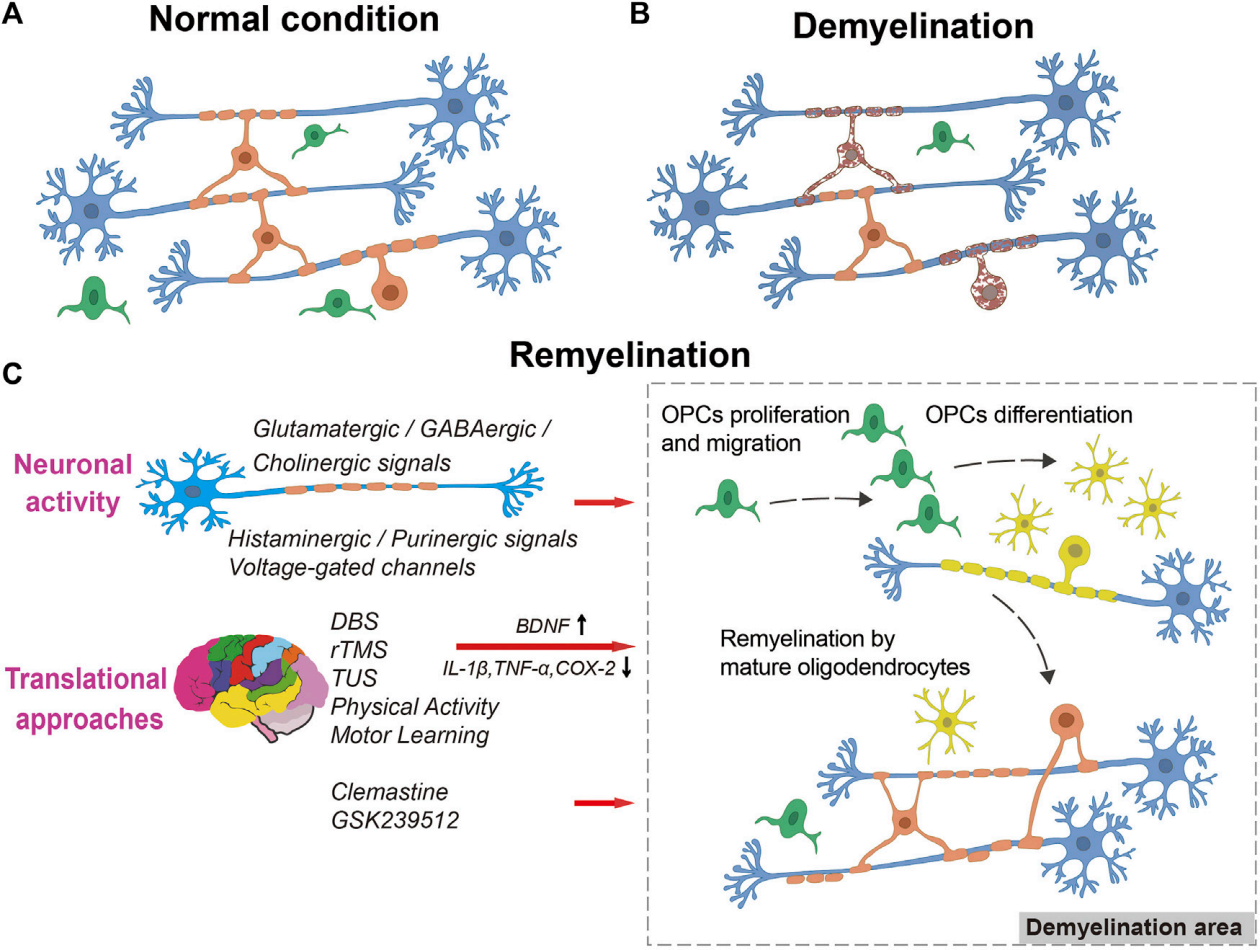
The process of remyelination. A diagram illustrating myelination in normal condition **(A)**, demyelination **(B)**, and remyelination **(C)**, showing OPC proliferation, migration into the lesion, differentiation into myelinating oligodendrocytes, and remyelination of naked axons by neuronal activity or translational approaches stimulation. Blue: neurons, Green: OPCs, Yellow: myelinating oligodendrocytes, Orange: mature oligodendrocytes. Dark red with spotted shape: dying oligodendrocytes.

Remyelination is a crucial process for restoring the function of nerves following damage. It involves the proliferation, migration, and differentiation of OPCs, which eventually wrap around axons to create new myelin sheaths (Figure1) (Smith et al., 1979; Horner and Gage, 2000; Mei et al., 2016).

Studies have shown that neuronal activity plays an essential role in myelin formation and remyelination. Previous studies indicated that electrical stimulation of neuronal activity promotes OPCs proliferation and myelination *in vitro* (Demerens et al., 1996; Bergles and Richardson, 2015). In contrast, inhibiting neuronal action potential generation using tetrodotoxin impeded the progression of myelination. Recent advances in biomolecular and genetic technologies have revealed that the precise activation and transmission of neurotransmitters within neurons have positive effects on myelin formation. Activation of cortical neurons promotes the proliferation and differentiation of OPCs, and it has even been shown to improve mobility in healthy mice (Demerens et al., 1996; Gibson et al., 2014; Pan and Chan, 2021). Similar evidence has been observed in both humans and mice, that the activation of neurons through enriched environments and complex motor skills hastens the formation of newborn myelin and myelin ultrastructure in white matter (McKenzie et al., 2014; Bacmeister et al., 2020; Bacmeister et al., 2022; Nicholson et al., 2022). In addition, the knockout of myelin regulatory factor (MYRF) in OPCs resulted in reduced motor learning ability and restraint of newborn myelin formation (McKenzie et al., 2014; Xiao et al., 2016). With 7 weeks of motor learning training, MYRF and Myelin Basic Protein (MBP) expression in wild-type mice increased by 1.4-fold, emphasizing the critical role of neurons in myelin genesis (Yoon et al., 2016). These studies provide the foundation for investigating the dynamic interactions of signaling pathways between neurons and oligodendrocytes.

Pharmacological blockade of neuronal activity in the demyelination model led to an increase in the number of OPCs within the lesions, but also a decreased proportion of differentiated oligodendrocytes, which impairs the regeneration of myelin *in vitro* (Gautier et al., 2015). Recent *in vivo* experiments demonstrated the neuroprotective effect of neuronal activity on remyelination (Ortiz et al., 2019; Zhou et al., 2022; Deng et al., 2023). Repetitive transcranial magnetic stimulation (rTMS) and transcranial ultrasound stimulation (TUS), non-invasive methods for controlling neuronal activity, encouraged OPC survival and differentiation, which aided in myelin repair and the restoration of cognitive function (Darmani et al., 2022). Precise modulation of neurons in the cerebral cortex utilizing optogenetics and chemogenetics promoted OPCs proliferation and differentiation and contributed to extensive remyelination, recovery of nerve conduction, and cognitive impairment in chronic hypoperfusion and lysophosphatidylcholine (LPC)-induced demyelination model. These effects were mediated through the Wnt2 pathway and neuron-OPC functional synapses. These findings provide novel insights into clinical treatments for demyelinating diseases via precise regulation of neuronal activity. However, it is uncertain whether remyelination is entirely dependent on neuronal activity since it may also affect other neurons and glia cells, not just oligodendrocytes and remyelination (Maas and Angulo, 2021). Further investigation is needed to identify the mechanisms involved in the interactions between neuronal activity and remyelination. In Table 1, the effects and molecular mechanisms of remyelination *in vitro* and *in vivo* by neurotransmitters and neuromodulators known to regulate neuronal activity are summarized.

**TABLE 1:** Effects and molecular mechanisms of neuronal activity on remyelination.

	Targets	*In Vitro*	*In Vivo* demyelination models
Glutamate	General glutamatergic receptors	Proliferation↓ (Spitzer et al., 2016)	Proliferation↑ and myelination↑ in cerebral hypoperfusion model (Zhou et al., 2022)
			Differentiation↑ and myelination↑ in chronic ischemia/LPC-induced model (Ortiz et al., 2019; Deng et al., 2023)
	AMPA receptors	Proliferation↓ and differentiation↓ (Fannon et al., 2015)	Proliferation↑ and myelination↑ in ethidium bromide-induced model (Gautier et al., 2015)
		Differentiation↑ and myelination↑ (Kukley et al., 2007; Barron and Kim, 2019)	Survival↑ and myelination↑ with GluA2/3/4 deficient mice (Kougioumtzidou et al., 2017)
	NMDA receptors	Myelination↓ (Salter and Fern, 2005; Christensen et al., 2016; Macrez et al., 2016)	Myelination↑ in EAE, ethidium bromide/stroke-induced model (Lundgaard et al., 2013; Doyle et al., 2018)
			Myelination↓ in EAE model (Macrez et al., 2016)
GABA	GABAA receptors	Proliferation↑ and differentiation↓ (Hamilton et al., 2017)	Proliferation↓ and myelination↑ in chronic neonatal hypoxia-induced model (Zonouzi et al., 2015; Bai et al., 2021)
	GABAB receptors	Myelination↑ (Serrano-Regal et al., 2022)	Differentiation↑ and myelination↑ in LPC-induced model (Serrano-Regal et al., 2022)
			Myelination↑ in MS patients (Cawley et al., 2015)
Acetylcholine	mAChRs	Proliferation↑ (Fields et al., 2017)	Differentiation↓ and myelination↓ in EAE/cerebral hypoxic model (Deshmukh et al., 2013; Cree et al., 2018)
		Differentiation↓ and Myelination↓ (De Angelis et al., 2012; Deshmukh et al., 2013)	
	nAChRs	Differentiation↑ (Imamura et al., 2015)	Myelination↑ in EAE model (Piovesana et al., 2022)
			Myelination↓ in EAE model/nAChR (α9/α10) knockout mice (Liu et al., 2019)
Histamine	H1 receptors		Myelination↓ in EAE model with H1 receptor knockout mice (Dimitriadou et al., 2000; Ma et al., 2002)
	H2 receptors		Differentiation↓ and myelination↓ in cerebral hypoxic model with Hrh2fl/fl; CNPase-Cre mice (Jiang et al., 2021a)
			Myelination↑ in EAE model with H2 receptor knockout mice (Saligrama et al., 2014)
	H3 receptors		Myelination↑ in EAE model with H3 receptor knockout mice (Teuscher et al., 2007; Shi et al., 2017)
			Myelination↓ in EAE model (Imeri et al., 2021)
	H4 receptors		Myelination↑ in EAE model (Ballerini et al., 2013)
	HDC		Myelination↑ in EAE model with HDC knockout mice (Saligrama et al., 2013)
Adenosine	General	Differentiation↑ and myelination↑ (Stevens et al., 2002)	
	P1 receptors		
	A2a/A2b receptors	Differentiation↓ (Coppi et al., 2013; Coppi et al., 2020)	
	A1 receptors		Myelination↓ in brain hypoxia model (Cherchi et al., 2021b)
	A3 receptors	Apoptosis↑ (Cherchi et al., 2021b)	
ATP/ADP	P2X7 receptors		Myelination↓ in EAE/cerebral ischemia model (Domercq et al., 2010; Illes, 2020)
			Overexpressed in the brains of patients with demyelination (Rivera et al., 2021)
K^+^	Kir4.1	Proliferation↑ (Rivera et al., 2021)	Differentiation↑ and myelination↑ in cerebral ischemia model (Song et al., 2018)
		Myelination↑ (Maldonado et al., 2013)	
	Kv1.3	Proliferation↑ (Rivera et al., 2021)	Proliferation↓ and differentiation↓ in Interleukin 17-induced model (Liu et al., 2021)
		Proliferation↓ and differentiation↓ (Liu et al., 2021)	
	Kv1.6	Proliferation↓ (Rivera et al., 2021)	
Ca^2+^	Cav1.2	Myelination↑ (Li et al., 2020)	Myelination↑ in cuprizone-induced model with Cav1.2 defective mice (Santiago Gonzalez et al., 2017; Paez and Lyons, 2020)
	PDE7	PDE7 inhibitors induce survival↑, differentiation↑ and myelination↑. (Medina-Rodriguez et al., 2013; Zorn and Baillie, 2023)	PDE7 inhibitors induce myelination↑ in EAE/cuprizone/LPC-induced model (Martin-Alvarez et al., 2017; Medina-Rodriguez et al., 2017)

### 2.1 Molecular mechanisms of glutamatergic signaling pathways

OPCs are distinguishable from mature oligodendrocytes and other types of glial cells, as they contain voltage-gated potassium (K^+^), sodium (Na^+^), and calcium (Ca^2+^) channels on their cell surface that can initiate action potentials (Paez et al., 2009). OPCs in the hippocampus can receive projections of glutamatergic neuron fibers, respond to nerve impulses, and generate action potentials (Bergles et al., 2000; Gautier et al., 2015). The synaptic connections between glutamatergic neurons and OPCs play a critical role in myelin formation, and synaptic vesicle release has been shown to affect the myelination process. Inhibition of glutamate release from neurons resulted in a significant decline of approximately 39% in myelination (Mensch et al., 2015). Neuronal activity in cultured neurons stimulated the synaptic release of glutamate, which increased the myelination of axons. Conversely, inhibition of vesicle release or glutamate receptor activity decreased myelination (Li et al., 2013). With the development of neuronal precision control technology, *in vivo* optogenetic and pharmacogenetic stimulations of glutamatergic neurons in mice have been demonstrated to boost OPCs proliferation, differentiation in white matter, and contribute to the formation of thicker myelin (Gibson et al., 2014; Mitew et al., 2018; Deng et al., 2023). Additionally, activating cortical glutamatergic neurons with optogenetics in the LPC-induced demyelination model and cerebral hypoperfusion enhanced OPCs proliferation and differentiation in regions of demyelination, leading to remyelination and cognitive function recovery (Ortiz et al., 2019; Zhou et al., 2022). Furthermore, functional synaptic connections between glutamatergic neurons and OPCs, marked by synaptic markers VgluT1 and VgluT2, were formed in areas of demyelination injury (Etxeberria et al., 2010; Gautier et al., 2015; Zhou et al., 2022). These studies indicated that the precise control of the glutamatergic neuron-OPC neuronal circuit to increase functional synaptic connections is necessary to protect against demyelination. However, the functional synaptic connections between OPCs and glutamatergic neurons were significantly altered in the LPC-induced demyelination injury models, resulting in a lack of VGlut1 puncta and neuron-OPC synaptic connections, especially in proliferating OPCs (Sahel et al., 2015). After reaching their proliferation peak, OPCs obtained synaptic connections from neurons during the subsequent differentiation and remyelination stages. These findings further support the notion that elevated glutamate neuronal activity positively impacts remyelination, although the specific mechanisms and processes involved in glutamatergic neuron-OPC functional synapses in this neuroprotective effect require further validation.

Glutamate receptors such as AMPA and NMDA are expressed on the cell membranes of OPCs, and glutamatergic signaling pathways are known to regulate OPCs development and myelin regeneration via regulation of these receptors. There have been multiple studies that revealed the signaling pathway mechanisms between glutamatergic neurons and OPCs, which improve the formation of myelin and remyelination (Krasnow and Attwell, 2016; Spitzer et al., 2016; Doyle et al., 2018). AMPA receptors are identified as the dominant receptor involved in remyelination. Gautier et al. reported that electrical stimulation of glutamatergic neurons, which senses neuronal activity by AMPA receptors, increases OPCs proliferation and promotes remyelination in demyelination lesions induced by ethidium bromide (Gautier et al., 2015). Actually, the role of AMPA receptors in OPCs proliferation is still controversial. In contrast to those findings, studies have reported that blocking AMPA receptors activity by pharmacological means or genetic knockout of subunits GluA2/3/4 does not appear to significantly impede OPCs proliferation *in vivo* or *in vitro* (Fannon et al., 2015; Kougioumtzidou et al., 2017). The AMPA receptors consistently exhibit significant effects on the later stages of myelin regeneration. Excitation of glutamatergic neurons and subsequent release of glutamate from activated axons led to OPCs depolarization and AMPA receptor activation on oligodendrocytes, which contribute to the positive influence of OPCs differentiation and myelination (Kukley et al., 2007; Barron and Kim, 2019). Conversely, blocking AMPA receptor activity arrested OPCs differentiation and subsequent remyelination (Gautier et al., 2015; Chen et al., 2018).

NMDA receptors are another key receptor involved in glutamatergic signaling pathways. Research has shown that excitation of NMDA receptors in OPCs, which are dependent on glutamatergic signaling, is essential for accelerating and improving myelin formation, while blockade of NMDA receptors with MK-801 results in the suppression of myelin formation (Lundgaard et al., 2013). The authors also reported that remyelination following white matter injury is reliant on NMDA receptors *in vivo*. Inhibiting NMDA receptors with MK-801 after demyelination led to a significant decrease in the number of myelinated axons and the thickness of the myelin sheath (Lundgaard et al., 2013). In addition, in both rat and mouse models of stroke-induced demyelination, stimulation of glutamatergic neurons resulted in the release of glutamate into myelin-wrapped axons and the activation of myelin NMDA receptors, which can protect myelin basic proteins from ischemic damage (Doyle et al., 2018). NMDA receptors maybe greatly correlated with OPCs differentiation and remyelination. These receptors were found to be rapidly upregulated at 14 days following demyelination injury in differentiating OPCs, implying their important roles in the differentiation phase of myelin repair (Salter and Fern, 2005; Gautier et al., 2015). However, it is worth noting that both the myelin and axonal structures underwent damage after a 3-h activation of NMDA receptors in dorsal roots incubation (Christensen et al., 2016). Additionally, the authors discovered that pretreatment with NMDA receptor antagonists can attenuate the pathological changes in demyelination disorders (Micu et al., 2006; Macrez et al., 2016). In light of the above conflicting data, further studies are imperative to ascertain whether NMDA receptors can indeed have beneficial effects on remyelination. In general, the glutamatergic signaling pathways exhibit complex actions in OPCs development and produce varying effects on myelin generation and remyelination stages.

### 2.2 Molecular mechanisms of GABAergic signaling pathways

OPCs express both the GABAA and GABAB receptors, which allow for GABAergic inhibitory and glutamatergic excitatory regulation (Zonouzi et al., 2015). Modulation of GABA receptors and GABAergic signaling pathways is implicated in the regulation of OPCs development, myelination, and remyelination (Bai et al., 2021; Serrano-Regal et al., 2022). In mouse cerebral slices, the presence of endogenous GABA elevated the rate of OPCs and decreased their maturation, an effect that could be nullified by the administration of gabazine, an antagonist of the GABAA receptor (Hamilton et al., 2017). These findings suggest that GABAA receptor-mediated signaling pathways can obstruct myelin synthesis. In contrast, studies have found that GABAA receptor-mediated signaling pathways are downregulated in OPCs in brains under chronic hypoxia and are associated with increased OPCs proliferation, disrupted initiation and progression of oligodendrocytes, and anomalous myelination (Zonouzi et al., 2015). Thus, impeding the metabolism or uptake of GABA can facilitate the differentiation of OPCs into mature oligodendrocytes, which facilitates myelin synthesis by improving the response to hypoxia. This difference in the role of GABAA receptor-mediated signaling pathways during myelination may be due to distinct physiological and pathological conditions. GABAB receptors also play a crucial role in regulating myelin synthesis and the regeneration of myelin. Intracerebral injection of GABAB receptor antagonists in neonatal rats increased OPCs proliferation as well as decreased MBP expression and myelin production in the cingulate cortex (Pudasaini et al., 2022). Baclofen, a GABAB receptor-specific agonist commonly used as a muscle relaxant in MS patients, heightened remyelination by prompting GABA to amplify Myelin-associated glycoprotein (MAG) and MBP expression levels *in vitro* in optic nerve explants and cerebral slices (Serrano-Regal et al., 2022). Serrano-Regal and colleagues further assessed the effects of Baclofen treatment on remyelination *in vivo* using the LPC-triggered demyelination mouse model. Their findings showed that such treatment encouraged the differentiation of OPCs and increased the prevalence of nascent axons in demyelinated lesions, thus indicating a beneficial impact on remyelination (Serrano-Regal et al., 2022). Studies on patient brains depicting progressive demyelination with MS revealed significantly decreased GABAergic signaling pathways and reduced GABA expression in the sensorimotor cortex and hippocampus relative to normal individuals (Cawley et al., 2015). Furthermore, lower GABA levels in the sensorimotor cortex were associated with diminished limb movement, and decreased GABA expression may indicate disruptions in physical abilities and neurodegenerative mechanisms. The findings of this study underscore the significance of regulating GABAergic signaling pathways as a promising prevention approach for neurodegenerative diseases involving demyelination. Overall, GABAergic receptors and signaling pathways play a vital role in promoting myelin synthesis and facilitating remyelination.

### 2.3 Molecular mechanisms of cholinergic signaling pathways

The involvement of acetylcholine (ACh) in myelin formation has been extensively reported. However, the specific impacts of cholinergic neurons and cholinergic signaling pathways on oligodendrocytes are yet to be clearly defined. Several investigations have substantiated the presence of ACh receptors in oligodendrocytes. Additionally, these studies have validated that the activation of signaling pathways in OPCs and other cells related to the oligodendrocyte lineage is induced by ACh receptors (Takeda et al., 1995; Larocca and Almazan, 1997). The oligodendrocytes express two types of ACh receptors, including muscarinic acetylcholine receptors (mAChRs) and nicotinic acetylcholine receptors (nAChRs) (Bernardini et al., 1999; De Angelis et al., 2012). The transcriptional regulation of cholinergic signaling pathways, activated by Ach receptors, may influence oligodendrocyte development and function. mAChRs, especially M1, M3, and M4 subtypes, were highly expressed on OPCs, suggesting that mAChRs are involved in the initial phases of oligodendrocyte development (Ragheb et al., 2001; De Angelis et al., 2012). According to Fields et al. revealed that pharmacological receptor antagonists can reduce the level of phosphorylated CREB and c-fos expression in OPCs and stimulate OPCs proliferation through the activation of cholinergic pathways mediated by mAChRs (Fields et al., 2017). Muscarinic signaling suppressed OPCs differentiation and myelin formation, and it caused a drop in MBP expression, as shown by further studies (De Angelis et al., 2012). Another research group discussed the utilization of the mAChR antagonist benztropine to accelerate OPCs maturation, resulting in a favorable outcome in alleviating demyelination in both *in vitro* and *in vivo* experiments (Wessler et al., 1999; Deshmukh et al., 2013). Additionally, previous studies have disclosed the presence of multiple subtypes of nAChRs in OPCs, localized in regions including the hippocampus and corpus callosum, which exert an impact on OPCs differentiation (Velez-Fort et al., 2009; Imamura et al., 2015; Imamura et al., 2020). The cholinergic anti-inflammatory system regulates internal immunological homeostasis and excessive immune inflammatory responses. Demyelination disorders have been found to exhibit defects in the anti-inflammatory signaling pathways of Ach that are mediated by nAChRs. The study conducted by Piovesana et al. demonstrated that applying of an acetylcholinesterase inhibitor resulted in the relief of neuroinflammation and demyelination induced by Experimental Autoimmune Encephalomyelitis (EAE), as well as an improvement in cognitive behavior deficits (Piovesana et al., 2022). Correspondingly, this neuroprotective effect was diminished by the treatment of α7nAChR antagonists. Nicotine administration or the use of nAChR α9/α10 knockout mice greatly decreased the severity of EAE and postponed the onset of disease symptoms. This study also revealed a decrease in immune cell infiltration, reactive oxygen species levels, and demyelination in the spinal cord and brain of nAChR α9/α10 knockout mice, indicating that nAChRs may play immunomodulatory roles in autoimmune demyelinating disorders (Liu et al., 2019). In addition, a non-selective antimuscarinic drug prevented demyelination-related schizophrenia-like behavior in a cuprizone mouse model and recovered myelin repair (Li et al., 2015). Through its specific actions on mAChR of OPCs, antimuscarinic effects dramatically aided OPCs differentiation and myelin production, reduced ultrastructural myelin degradation, and improved functional recovery after hypoxic brain injury (Cree et al., 2018).

Acetylcholinesterase inhibitors have been shown to improve the myelin regeneration in demyelination diseases, including MS and its animal models. Imamura et al. showed that employing the acetylcholinesterase inhibitor donepezil pharmacologically improves differentiation and maturation of OPCs derived from neural stem cells without affecting their proliferation or survival (Imamura et al., 2015; Imamura et al., 2017). The administration of donepezil resulted in a significant upregulation of critical proteins associated with myelin, such as MAG, Myelin proteolipid protein (PLP), and MBP. The promotion of OPCs maturation by donepezil was obviously inhibited by the nAChR antagonist Mecamylamine. However, a recent study demonstrated that inhibiting the cholinergic signaling pathways might reduce the speed of myelin conduction and myelin thickness in the corpus callosum, leading to the failure of myelin synthesis (Palma et al., 2022). The preexisting data is consistent with the involvement of cholinergic signaling pathways in cognitive dysfunction and demyelination-related white matter diseases. Regulation of cholinergic signaling pathways may offer possible therapeutic treatments to promote myelin regeneration and white matter recovery, but there is still debate in this field.

### 2.4 Molecular mechanisms of histaminergic signaling pathways

Histamine is a pervasive inflammatory mediator implicated in the pathophysiology of diverse allergy, autoimmune, inflammatory, and neurological disorders (Hu and Chen, 2017; Volonte et al., 2022). Histamine signaling has been reported to regulate the differentiation of OPCs, attenuate demyelination, and improve myelin repair, particularly in MS (Panula and Nuutinen, 2013; Jiang et al., 2021a; Amadio et al., 2022). The effects of histaminergic signaling have been noted to be both beneficial and detrimental through its immunomodulatory properties or direct regulation of histaminergic H1-H4 receptors in MS (Passani and Blandina, 2011; Passani and Ballerini, 2012; Palma et al., 2022). Histamine has the potential to alter the permeability of the blood-brain barrier and increase the number of infiltrating cells in the central nervous system, resulting in detrimental neuroinflammation (Volonte et al., 2022). In EAE model mice, the H1 receptors have the potential to speed up the occurrence of immunological inflammation, demyelination, and behavioral abnormalities (Ma et al., 2002). The most recent research found that H2 receptors, like H1 receptors, has a pro-pathogenic effect through decreasing OPCs maturation following hypoxic brain injury (Jiang et al., 2021a). H2 receptors also exhibited a neuroprotective effect by attenuating demyelination, but it also induced a positive suppression of the immunological inflammatory response (Saligrama et al., 2014). The H3 receptors act as an auto-receptor, modulating the activity of histaminergic neurons and regulating the synthesis and secretion of histamine (Passani and Blandina, 2011). The application of the highly selective histamine H3 receptor agonist Immethridine slowed the progression of the disease by reducing the expression of inflammatory cytokines such as TNFα and IFN-γ in an EAE model (Teuscher et al., 2007; Shi et al., 2017). Recent researches using methylene amine and two novel piperidine compounds as histamine H3 receptor antagonists validated the beneficial effects of MS treatment (Imeri et al., 2021). In accordance with the conclusions, demyelination, inflammatory infiltration, and the EAE clinical score were all decreased. Moreover, H4 receptors have a neuroprotective effect comparable to that of H3 receptors. H4 receptors deletion or use of the H4 receptors antagonist JNJ7777120 led to increased spinal cord inflammation and IFN- levels, which hindered remyelination (Ballerini et al., 2013; Volonte et al., 2022).

Histidine decarboxylase (HDC) is capable of regulating histaminergic signaling pathways and the release of endogenous histamine. Musio et al. showed that endogenous histamine has an inhibitory effect on immunological damage and myelin breakdown in the central nervous system, promoting the therapeutic efficacy of EAE (Musio et al., 2006). Histamine-deficient HDC knockout mice exhibited more severe neuropathy and EAE symptoms. The transcription of genes associated with oligodendrocytes and myelin production in the basal ganglia was altered in HDC knockout mice with defective histaminergic signaling pathways, which resulted in white matter damage and abnormal mental states (Saligrama et al., 2013). A first-generation antihistamine, Clemastine, showed benefits in promoting oligodendrocyte differentiation and has been evaluated as a remyelinating therapy for multiple sclerosis in clinical trials (Green et al., 2017). The detailed information on Clemastine is in Part 3.5. Serum levels of histamine and its precursor histidine were lower in MS patients than in healthy individuals, especially in women with clinically significant symptoms, indicating that histaminergic signaling pathways play a significant role in MS-related symptoms (Rafiee Zadeh et al., 2018; Loy et al., 2019). Nevertheless, an inverse correlation between histamine levels in the central nervous system and serum has been observed in MS patients by other investigators (Cacabelos et al., 2016a; Cacabelos et al., 2016b). Therefore, additional research is required to elucidate the specific roles of histamine and histaminergic signaling pathways.

### 2.5 Molecular mechanisms of purinergic signaling pathways

Purinergic signaling pathways have been shown to influence oligodendrocyte growth, myelin formation, and remyelination in both healthy and pathological conditions (Rivera et al., 2016; Butt et al., 2019). Adenosine and ATP are acknowledged as immune function modulators, functioning as activators and chemotactic signals for diverse immune cells (Welsh and Kucenas, 2018). Purines serve as neurotransmitters and facilitate the transmission of neural signals through the activation of purinergic receptors. Oligodendrocytes predominantly express adenosine-binding P1 receptors and ATP- and ADP-binding P2 receptors (Cheng et al., 2023). P1 receptors are subdivided into four distinct types, namely, A1, A2a, A2b, and A3, all of which are G protein-coupled receptors. A1 and A3 exert inhibitory effects on adenylate cyclase, resulting in a reduction of intracellular cyclic adenosine monophosphate (cAMP) levels. Conversely, A2a and A2b stimulate adenylate cyclase, leading to an elevation in intracellular cAMP concentrations (Welsh and Kucenas, 2018). P2 receptors are classified into ionotropic P2X (P2X1-7) or metabotropic P2Y(P2Y1, P2Y2, P2Y4, P2Y6, and P2Y11-14) subtypes.

Adenosine is involved in the regulation of OPCs development through a dual mechanism. The administration of adenosine and the P1 receptor agonist NECA in co-cultures of OPC-DRG cells exhibited a notable reduction in OPC proliferation in a concentration-dependent manner, while concurrently promoting OPC differentiation and myelin regeneration (Stevens et al., 2002; Welsh and Kucenas, 2018). However, recent studies have reported that the maturation of OPCs can be hindered by A2a receptor and A2b receptor agonists through the inhibition of potassium currents, whereas cell proliferation is unaffected (Coppi et al., 2013; Coppi et al., 2020; Cherchi et al., 2021b). Adenosine is frequently thought to have a protective effect in white matter disease models. Several studies have indicated that the absence of A1 receptors and A2a receptors in mice can intensify white matter damage in EAE models because of the anti-inflammatory properties of adenosine on immune cells and microglia (Tsutsui et al., 2004; Yao et al., 2012; Welsh and Kucenas, 2018). The mechanism of these neuroprotective effects may be attributed to immunomodulation. Contrary to the newborn rat brain hypoxia model, however, treatment with A1 receptor agonists reduces the volume of white matter, causes ventricular enlargement, and lowers MBP expression (Cherchi et al., 2021b). Furthermore, an additional research group has disclosed that the activation of A3 receptors by adenosine may induce oligodendrocyte apoptosis in optic nerve and white matter ischemic injury (Gonzalez-Fernandez et al., 2014). The diverse functions of adenosine on oligodendrocytes and immune cells under distinct pathophysiological conditions are essential for the treatment of demyelination diseases.

P2X7 receptors are the purinergic receptors that have been investigated the most. According to available research, high levels of ATP that constantly activate P2X7 receptors in oligodendrocytes cause oligodendrocyte death and myelin loss in demyelination disorders *in vivo* (Illes, 2020). In addition, P2X7 receptor antagonists were noted to be very effective in preventing oligodendrocyte apoptosis and to be implicated in the modulation of white matter injury in cerebral ischemia (Domercq et al., 2010). In a clinical study, Oyanguren-Desez et al. discovered a gain-of-function mutation of the P2X7 receptors in patients with multiple sclerosis (Oyanguren-Desez et al., 2011). This mutation resulted in an elevated inward flow of cytotoxic calcium. P2X7 receptors were also found to be overexpressed in the brains of patients with white matter atrophy and myelin loss compared to healthy controls (Rivera et al., 2021). Nonetheless, since many neuronal cells and immune cells in the periphery express P2X7 receptors, the effects of P2X7 receptor antagonists cannot be ascribed to direct effects on oligodendrocytes or immunomodulation.

Moreover, changes in the expression of P2Y2 and P2Y12 receptors on oligodendrocytes were seen in the demyelination models, but it is yet unknown if these changes have beneficial or detrimental consequences on myelin (Amadio et al., 2010; Welsh and Kucenas, 2018; Cherchi et al., 2021a).

### 2.6 Molecular mechanisms of voltage-gated channels

Different voltage-gated ion channels are expressed by OPCs in gray and white matter. These include voltage-gated K^+^ channels, Na^+^ channels, and Ca^2+^ channels that regulate the OPCs cell cycle and function (Liu et al., 2021). Overexpression of K^+^ channel protein subunits Kir4.1 and Kv1.3 stimulated OPCs proliferation *in vitro*, whereas overexpression of Kv1.6 inhibited mitogen-induced cell cycle progression, indicating the involvement of K^+^ channels in regulating OPCs proliferation (Larson et al., 2016). Kir4.1 modulated OPCs proliferation and differentiation in response to extracellular K^+^ variations during neuronal activity (Maldonado et al., 2013). Increased extracellular membrane K^+^ levels activated Kir4.1 channels in OPCs, which could speed up their proliferation and remyelination following ischemic cerebral injury (Song et al., 2018). Correspondingly, Kir4.1 channel defects in OPCs aggravated myelin protein loss and sheath ultrastructure disruption. Kv1.3, an additional prevalent voltage-gated K^+^ channel on oligodendrocytes, displayed higher levels of expression in an inflammatory model of myelin injury induced by Interleukin 17 *in vitro* and *in vivo* experiments (Liu et al., 2021). Interleukin 17 accelerated myelin damage by altering Kv1.3 currents and blocking AKT phosphorylation, thereby inhibiting OPCs proliferation and differentiation. The preceding findings suggest that K^+^ channels may be a potential intervention target for myelin impairment.

The function of the Na^+^ channel remains uncertain. Tong et al. provided evidence that GABA induces activation of GABAA receptors, which in turn causes a sustained elevation of Na^+^ currents in the OPCs, resulting in Ca^2+^ influx via the Na^+^/Ca2^+^ exchanger. This particular Ca^2+^ signaling pathway was linked to OPCs migration and myelination (Tong et al., 2009). GABA and glutamate transmitters are released by neurons, activating AMPA and GABA receptors in the OPCs. Sun and colleagues found that activation of the majority of AMPA and GABA receptors is correlated with an increase in intracellular Ca^2+^ signaling (Sun et al., 2016). With Ca^2+^ signaling pathways, these activated receptors communicate with neurons and OPCs to generate myelin sheaths (Li et al., 2020). The frequency of the Ca^2+^ signal transient was found to correspond with the extension of the myelin sheath when it was caused by high-frequency stimulation (Baraban et al., 2018; Krasnow et al., 2018). In contrast, low-frequency stimulation and less Ca^2+^ release shorten the myelin sheath. In a cuprizone demyelination model, animals with a specific deletion of Ca^2+^ channel subtype Cav1.2 on OPCs suffered remyelination disorder (Santiago Gonzalez et al., 2017). Cav1.2 defective mice revealed a reduction in OPCs number, myelin proteins MBP synthesis, myelinated axons, and myelin thickness, which indicated that Ca^2+^singaling activation and Ca^2+^ inflow of OPCs were needed for myelin repair (Paez and Lyons, 2020). On the whole, the modulation of voltage-gated ion channels expression and activity is a promising therapeutic strategy for promoting OPCs proliferation and differentiation and improving remyelination after injury.

Along with the aforementioned neural signaling pathways, small-molecule agents like Phosphodiesterase 7(PDE7) inhibitors can also improve myelination and remyelination by affecting neuronal activity and immunomodulation (Huang et al., 2023). The study conducted by Zorn and Baillie et al. found that increased PDE7 expression and a subsequent decrease in cAMP levels were substantially correlated with MS disease development and symptoms in animal models (Zorn and Baillie, 2023). PDE7 inhibitors VP1.15 and VP3.15 enhanced motor function and alleviated demyelination and disease pathology in EAE, cuprizone, and LPC-induced demyelination models by increasing intracellular cAMP and reducing pro-inflammatory cytokines (Martin-Alvarez et al., 2017; Medina-Rodriguez et al., 2017). PDE7 inhibitors have also been demonstrated in *ex vivo* studies to prevent neuronal degeneration, encourage OPCs survival, differentiation, and remyelination (Medina-Rodriguez et al., 2013; Zorn and Baillie, 2023). Therefore, in the future, treating demyelination diseases by targeting PDE7 might be an innovative strategy.

## 3 Translational approaches and drugs

Modulation of neuronal signaling pathways that directly impacts on neuronal activity could promote OPC survival, proliferation, and differentiation, thus making neuronal activity as a potent extrinsic regulator of remyelination in demyelination disease. Neuronal activity can be modulated by invasive way such as deep brain stimulation (DBS) and noninvasive ways like repetitive rTMS, TUS and behavioral training (Darmani et al., 2022). In addition to directly stimulating neuronal activity, alternative pathways can be sought, such as medicines that modulated neuronal axonal signaling during OPCs differentiation and remyelination.

### 3.1 DBS

Currently, DBS has been FDA-approved for the treatment of several conditions, including Parkinson’s disease, dystonia, essential tremor, obsessive-compulsive disorder, and medically refractory epilepsy. Although the underlying mechanisms were largely unknown, DBS was well tolerated and improved the mean tremor rating scale scores of multiple sclerosis patients at 6 months (Oliveria et al., 2017). Memory and cognitive ability are dependent on regulating the fornix, a white matter tract in the Papez circuit. Lee et al. have conducted clinical trials in phases 1 and 2, which focus on fornical DBS as a treatment for disorders of the nervous system and schizophrenia that involve white matter (Lee et al., 2019). However, invasive methods of brain stimulation may result in lesions of the white matter and an impairment in verbal fluency (Costentin et al., 2019).

### 3.2 rTMS

It is reported that low intensity rTMS in an intermittent theta burst stimulation pattern upregulated oligodendrocytes survival and promoted myelination. Four weeks of daily rTMS has been demonstrated to enhance oligodendrocyte myelination in both healthy adult mice and demyelinated mice, while also reducing depression-like symptoms and facilitating cognitive recovery in demyelinated mice (Cullen et al., 2019; Wang et al., 2021; Mooshekhian et al., 2022). Recently, a randomized controlled trial has been conducted to assess the safety and remyelination in multiple sclerosis patients (Makowiecki et al., 2022). But not only neuronal cells were affected by magnetic stimulation, glial progenitor cells could also be influenced directly. Gamma frequency low-field magnetic stimulation daily for five consecutive days *in vitro* was reported to promote the differentiation of OPCs (Dolgova et al., 2021). The underlying molecular mechanism of remyelination has been linked to elevated brain-derived neurotrophic factor (BDNF) levels and decreased Interleukin-1 beta (IL-1β) and Tumor necrosis factor alpha (TNF-α) levels during rTMS treatment (Zhao et al., 2019).

### 3.3 TUS

Ultrasound treatment was demonstrated to enhance remyelination in the LPC-induced demyelination model of MS by alleviating neuroinflammation, enhancing mature oligodendrocyte density, and promoting BDNF expression (Yang et al., 2022). TUS has higher spatial resolution and deeper penetration, which can induce short-term and long-term changes in neuronal excitability and spontaneous firing rates. Also, it has the ability to selectively target small subcortical structures compared to other non-invasive stimulation methods (Darmani et al., 2022). Another advantage of TUS is that it can be safely combined with MRI-related neuroimaging techniques such as diffusion tensor imaging, which could reflect the effects of TUS treatment on white matter fiber tracts. In all, it seems that TUS could serve as a potential treatment for demyelinated disorders that needs modulation of neuronal activity. But TUS is still in the research stage, and further clinical trials are needed to evaluate its safety and efficacy (Darmani et al., 2022).

### 3.4 Physical activity and motor learning

Motor learning, the progressive acquisition of a specific novel motor skill, showed influences on adaptive myelin formation in both the healthy and demyelinated central nervous system. While physical activity, a planned, structured, and repetitive movement of the body that expends energy and improves one’s fitness, could promote remyelination of the nervous system pathological process (Bloom et al., 2022). White matter integrity affects cognitive performance, and conversely, behavioral training could also promote white matter regeneration. Behavior-induced neuronal activity could have influences on circuit-specific changes in myelination (Bacmeister et al., 2022). Exercise and motor skills training contribute to remyelination. It has been reported that precisely timed motor learning following the commencement of remyelination enhances remyelination from newly generated and persisting oligodendrocytes, improving recovery from demyelinating injury (Bacmeister et al., 2020). In the LPC or cuprizone model of demyelination, voluntary exercise may inhibit demyelination or encourage remyelination (Jensen et al., 2018; Mandolesi et al., 2019). And physical exercise was also reported to have an influence on astrocytes polarization, which facilitated the clearance of myelin debris and promoted remyelination (Jiang et al., 2021b). Therefore, individualized behavioral therapies are being employed more frequently in clinical settings to improve motor function in myelin disease patients.

### 3.5 Clemastine

It has been demonstrated that Clemastine, a first-generation antihistamine and M1/M3 muscarinic receptor antagonist, may stimulate OPCs development and remyelination *in vitro*, in several myelin degeneration models, and in human cells (Li et al., 2015; Liu et al., 2016; Tong et al., 2022). In a randomized, controlled, double-blind, crossover trial, it was evaluated as a remyelinating therapy for multiple sclerosis. The trial was carried out in a single center and encompassed a cohort of 50 patients with relapsing multiple sclerosis with chronic demyelinating optic neuropathy on stable immunomodulatory therapy. The administration of Clemastine resulted in a reduction of the latency delay by 1.7 ms/eye, when the trial was analyzed as a crossover. Treatment with Clemastine was linked to fatigue, but no significant adverse events were observed. The findings revealed that myelin repair is possible even in the presence of extensive injury (Green et al., 2017).

### 3.6 GSK239512

H3 receptors are highly expressed in the presynaptic region of neurons containing histamine, and moderately expressed in oligodendrocytes (Chen et al., 2017). It can directly regulate the synthesis and release of neurotransmitters in the central nervous system, which are important for processes of cognition, sleep, and homoeostatic neuronal regulation. GSK239512 is a highly effective and capable H3 receptor antagonist/inverse agonist. It has been specifically designed for the purpose of addressing cognitive impairment in neurological disorders, as increasing acetylcholine release could be one mechanism (Wilson et al., 2013). GSK239512 is a highly effective and capable H3 receptor antagonist/inverse agonist that has been specifically designed for the purpose of addressing cognitive impairment in neurological disorders. The benefits of the treatment for patients diagnosed with relapsing-remitting multiple sclerosis were evaluated through a clinical trial. The trial was conducted in a single center and involved 131 patients with relapsing-remitting multiple sclerosis on stable immunomodulatory therapy. The primary outcome was normalization of magnetization transfer ratio (MTR) changes in gadolinium-enhancing and Delta-MTR lesions. The outcome showed GSK239512 treatment was associated with a modest yet favorable impact on remyelination (Schwartzbach et al., 2017; Wooliscroft et al., 2019). However, we cannot rule out the possibility that GSK239512 could directly act on oligodendrocytes. Because it has been demonstrated that H3R antagonists can accelerate the differentiation of isolated OPCs *in vitro* (Chen et al., 2017).

## 4 Conclusion

Remyelination occurs in various clinical disorders, but it does not contribute to full functional recovery. Several clinical trials have been conducted to enhance remyelination, but they have been unsatisfactory (Lubetzki et al., 2020). Recent evidence suggests that increased myelin formation and remyelination can be stimulated by neuronal activity, which can aid in the development of therapeutic strategies for demyelination diseases (Maas and Angulo, 2021). Pharmacological blockade of neuronal activity impairs myelin regeneration, while non-invasive methods for controlling neuronal activity encourage OPC survival and differentiation, aiding myelin repair and cognitive function restoration (Darmani et al., 2022).

OPCs development, myelin formation, and remyelination depend on glutamatergic signaling pathways. The synaptic connections between glutamatergic neurons and OPCs positively influence remyelination by regulating the expression of AMPA and NMDA receptors on OPCs. AMPA receptors exhibit significant effects on the later stages of myelin regeneration, while NMDA receptors are essential for accelerating and improving myelin formation (Gautier et al., 2015; Chen et al., 2018). However, the effects of NMDA receptors on myelin and axonal structures are conflicting, and further studies are necessary to ascertain their beneficial effects on remyelination (Christensen et al., 2016).

GABAergic signaling pathways refer to the regulation of cells in the brain known as OPCs. The GABAA receptor-mediated signaling pathways obstruct myelin synthesis, their impacts on myelination may alter due to normal and pathological circumstances (Zonouzi et al., 2015). GABAB receptors also play an important role in regulating myelin synthesis and remyelination, as evidenced by studies using GABAB receptor antagonists and agonists (Pudasaini et al., 2022; Serrano-Regal et al., 2022). Furthermore, lower levels of GABA in patient brains with neurodegenerative diseases involving demyelination suggest the importance of regulating GABAergic signaling pathways for prevention (Cawley et al., 2015).

The involvement of ACh in myelin formation and remyelination has been extensively studied, and the activation of cholinergic signaling pathways mediated by ACh receptors affect the development and function of oligodendrocytes. Myelin regeneration and OPC differentiation are inhibited by muscarinic signaling, whereas nicotinic signaling boosts OPC maturation (Imamura et al., 2020; Piovesana et al., 2022).

Histamine has been found to regulate the differentiation of OPCs and attenuate demyelination, leading to improved myelin repair. Activation or inhibition of histaminergic H1-H4 receptors can result in different effects on demyelination and immunological inflammation (Piovesana et al., 2022). Histaminergic signaling pathways contribute to MS-related symptoms, as MS patients have lower serum histamine and histidine levels than healthy people (Rafiee Zadeh et al., 2018; Loy et al., 2019).

Purinergic signaling pathways influence oligodendrocytes development, myelin formation, and remyelination in both healthy and pathological conditions. Purines act as neurotransmitters, while adenosine and ATP regulate the immune response. Oligodendrocytes express P1 and P2 receptors, which regulate OPCs development through a dual mechanism (Welsh and Kucenas, 2018; Cherchi et al., 2021b). Adenosine has a protective effect in white matter disease models, and its anti-inflammatory properties may be attributed to immunomodulation (Coppi et al., 2013; Coppi et al., 2020). P2X7 receptors are the most investigated purinergic receptors, and their effects on oligodendrocyte death and myelin loss can’t be traced back to direct effects on oligodendrocytes or changes in the immune system (Illes, 2020).

Voltage-gated ion channels in OPCs influence the cell cycle and remyelination. Kir4.1 and Kv1.3 overexpression promote OPCs proliferation and myelination regeneration, but Kv1.6 inhibits it (Song et al., 2018; Liu et al., 2021). Myelin repair also requires Cav1.2 activation and Ca^2+^ signal transients. Modulation of voltage-gated ion channels may improve OPC proliferation and remyelination following damage (Paez and Lyons, 2020).

Modulating neuronal activity can promote remyelination in demyelination disease, using invasive methods like DBS or noninvasive methods like rTMS, TUS, as well as physical activity and motor learning. In therapeutic settings, individualized behavioral therapies are utilized to improve motor function in myelin disease patients (Makowiecki et al., 2022). Except for rTMS and TUS, the underlying molecular mechanisms of exercise have also been linked to the increased expression of BDNF (Wrann et al., 2013). BDNF, a member of the neurotrophic family, was demonstrated to impact oligodendrocyte lineage cells throughout development (Vondran et al., 2010). Lack of BDNF restricted proliferation and differentiation of OPC in the demyelination model (VonDran et al., 2011; Fulmer et al., 2014; Tsiperson et al., 2015). And several studies revealed that MS patients have lower levels of BDNF in their serum or cerebrospinal fluid (Azoulay et al., 2005; Azoulay et al., 2008). BDNF could also alleviate neuroinflammation by downregulating Cyclooxygenase-2 (COX-2) and pro-inflammatory cytokines in microglia (Lai et al., 2018; Nociti and Romozzi, 2023). In clinical trials for the treatment of multiple sclerosis, Clemastine and GSK239512 have demonstrated encouraging results in increasing myelination (Wooliscroft et al., 2019; Tong et al., 2022). To evaluate the effectiveness and safety of these techniques, nevertheless, more studies and trials are required.

These recent investigations have emphasized the potential of neuronal activity to promote myelin synthesis and repair. The use of optogenetics and chemogenetics to modulate neuronal activity possesses high cellular selectivity and exact spatiotemporal responses, allowing for the precise excitation or inhibition of specific neurons (Deisseroth, 2015; Roth, 2016). Non-invasive techniques, including DBS, rTMS, and TUS, offer potential avenues for clinical translation as new therapeutic strategies for demyelination diseases (Darmani et al., 2022). In contrast to pharmacological methods directly manipulating oligodendrocytes, a novel mechanism of action promotes the establishment of functional synaptic connections between OPCs and neurons by precisely modulating neuronal circuits (Etxeberria et al., 2010; Sahel et al., 2015; Zhou et al., 2022). The interaction between neurons and oligodendrocytes is increasingly recognized as one of the essential components of remyelination.

In this review, we emphasize the need for further research to develop effective therapeutic strategies for enhancing remyelination and neuroprotection associated with demyelination. We focus on recent developments in neuronal activity and remyelination, as well as their associated molecule mechanisms of neural signaling pathways and advanced clinical methods, which may potentially contribute to the development of therapeutic approaches for demyelination diseases.

## References

[B1] Amadio S. Conte F. Esposito G. Fiscon G. Paci P. Volonte C. (2022). Repurposing histaminergic drugs in multiple sclerosis. Int. J. Mol. Sci. 23, 6347. 10.3390/ijms23116347 35683024 PMC9181091

[B2] Amadio S. Montilli C. Magliozzi R. Bernardi G. Reynolds R. Volonte C. (2010). P2Y12 receptor protein in cortical gray matter lesions in multiple sclerosis. Cereb. Cortex 20, 1263–1273. 10.1093/cercor/bhp193 19783848

[B3] Azoulay D. Urshansky N. Karni A. (2008). Low and dysregulated BDNF secretion from immune cells of MS patients is related to reduced neuroprotection. J. Neuroimmunol. 195, 186–193. 10.1016/j.jneuroim.2008.01.010 18329726

[B4] Azoulay D. Vachapova V. Shihman B. Miler A. Karni A. (2005). Lower brain-derived neurotrophic factor in serum of relapsing remitting MS: Reversal by glatiramer acetate. J. Neuroimmunol. 167, 215–218. 10.1016/j.jneuroim.2005.07.001 16083971

[B5] Bacmeister C. M. Barr H. J. Mcclain C. R. Thornton M. A. Nettles D. Welle C. G. (2020). Motor learning promotes remyelination via new and surviving oligodendrocytes. Nat. Neurosci. 23, 819–831. 10.1038/s41593-020-0637-3 32424285 PMC7329620

[B6] Bacmeister C. M. Huang R. Osso L. A. Thornton M. A. Conant L. Chavez A. R. (2022). Motor learning drives dynamic patterns of intermittent myelination on learning-activated axons. Nat. Neurosci. 25, 1300–1313. 10.1038/s41593-022-01169-4 36180791 PMC9651929

[B7] Bai X. Kirchhoff F. Scheller A. (2021). Oligodendroglial GABAergic signaling: More than inhibition. Neurosci. Bull. 37, 1039–1050. 10.1007/s12264-021-00693-w 33928492 PMC8275815

[B8] Ballerini C. Aldinucci A. Luccarini I. Galante A. Manuelli C. Blandina P. (2013). Antagonism of histamine H4 receptors exacerbates clinical and pathological signs of experimental autoimmune encephalomyelitis. Br. J. Pharmacol. 170, 67–77. 10.1111/bph.12263 23735232 PMC3764850

[B9] Baraban M. Koudelka S. Lyons D. A. (2018). Ca (2+) activity signatures of myelin sheath formation and growth *in vivo* . Nat. Neurosci. 21, 19–23. 10.1038/s41593-017-0040-x 29230058 PMC5742537

[B10] Barron T. Kim J. H. (2019). Neuronal input triggers Ca(2+) influx through AMPA receptors and voltage-gated Ca(2+) channels in oligodendrocytes. Glia 67, 1922–1932. 10.1002/glia.23670 31313856 PMC6771819

[B11] Bergles D. E. Richardson W. D. (2015). Oligodendrocyte development and plasticity. Cold Spring Harb. Perspect. Biol. 8, a020453. 10.1101/cshperspect.a020453 26492571 PMC4743079

[B12] Bergles D. E. Roberts J. D. Somogyi P. Jahr C. E. (2000). Glutamatergic synapses on oligodendrocyte precursor cells in the hippocampus. Nature 405, 187–191. 10.1038/35012083 10821275

[B13] Bernardini N. Levey A. I. Augusti-Tocco G. (1999). Rat dorsal root ganglia express m1-m4 muscarinic receptor proteins. J. Peripher Nerv. Syst. 4, 222–232. 10642090

[B14] Bloom M. S. Orthmann-Murphy J. Grinspan J. B. (2022). Motor learning and physical exercise in adaptive myelination and remyelination. ASN Neuro 14, 17590914221097510. 10.1177/17590914221097510 35635130 PMC9158406

[B15] Butt A. M. Papanikolaou M. Rivera A. (2019). Physiology of oligodendroglia. Adv. Exp. Med. Biol. 1175, 117–128. 10.1007/978-981-13-9913-8_5 31583586

[B16] Cacabelos R. Torrellas C. Fernandez-Novoa L. Aliev G. (2016a). Neuroimmune crosstalk in CNS disorders: The histamine connection. Curr. Pharm. Des. 22, 819–848. 10.2174/1381612822666151209150954 26648474

[B17] Cacabelos R. Torrellas C. Fernandez-Novoa L. Lopez-Munoz F. (2016b). Histamine and immune biomarkers in CNS disorders. Mediat. Inflamm., 2016, 1924603, 10.1155/2016/1924603 PMC4846752 27190492

[B18] Cawley N. Solanky B. S. Muhlert N. Tur C. Edden R. A. Wheeler-Kingshott C. A. (2015). Reduced gamma-aminobutyric acid concentration is associated with physical disability in progressive multiple sclerosis. Brain 138, 2584–2595. 10.1093/brain/awv209 26304151 PMC4643627

[B19] Chen T. J. Kula B. Nagy B. Barzan R. Gall A. Ehrlich I. (2018). *In vivo* regulation of oligodendrocyte precursor cell proliferation and differentiation by the AMPA-receptor subunit GluA2. Cell. Rep. 25, 852–861 e7. 10.1016/j.celrep.2018.09.066 30355492

[B20] Chen Y. Zhen W. Guo T. Zhao Y. Liu A. Rubio J. P. (2017). Histamine Receptor 3 negatively regulates oligodendrocyte differentiation and remyelination. PLoS One 12, e0189380. 10.1371/journal.pone.0189380 29253893 PMC5734789

[B21] Cheng R. D. Ren W. Luo B. Y. Ye X. M. (2023). The role of purinergic receptors in neural repair and regeneration after spinal cord injury. Neural Regen. Res. 18, 1684–1690. 10.4103/1673-5374.363186 36751780 PMC10154499

[B22] Cherchi F. Bulli I. Venturini M. Pugliese A. M. Coppi E. (2021a). Ion channels as new attractive targets to improve Re-myelination processes in the brain. Int. J. Mol. Sci. 22, 7277. 10.3390/ijms22147277 34298893 PMC8305962

[B23] Cherchi F. Pugliese A. M. Coppi E. (2021b). Oligodendrocyte precursor cell maturation: Role of adenosine receptors. Neural Regen. Res. 16, 1686–1692. 10.4103/1673-5374.306058 33510056 PMC8328763

[B24] Christensen P. C. Welch N. C. Brideau C. Stys P. K. (2016). Functional ionotropic glutamate receptors on peripheral axons and myelin. Muscle Nerve 54, 451–459. 10.1002/mus.25078 26872412

[B25] Coppi E. Cellai L. Maraula G. Pugliese A. M. Pedata F. (2013). Adenosine A₂A receptors inhibit delayed rectifier potassium currents and cell differentiation in primary purified oligodendrocyte cultures. Neuropharmacology 73, 301–310. 10.1016/j.neuropharm.2013.05.035 23770463

[B26] Coppi E. Cherchi F. Fusco I. Dettori I. Gaviano L. Magni G. (2020). Adenosine A(2B) receptors inhibit K(+) currents and cell differentiation in cultured oligodendrocyte precursor cells and modulate sphingosine-1-phosphate signaling pathway. Biochem. Pharmacol. 177, 113956. 10.1016/j.bcp.2020.113956 32251679

[B27] Costentin G. Derrey S. Gerardin E. Cruypeninck Y. Pressat-Laffouilhere T. Anouar Y. (2019). White matter tracts lesions and decline of verbal fluency after deep brain stimulation in Parkinson's disease. Hum. Brain Mapp. 40, 2561–2570. 10.1002/hbm.24544 30779251 PMC6865750

[B28] Cree B. A. C. Niu J. Hoi K. K. Zhao C. Caganap S. D. Henry R. G. (2018). Clemastine rescues myelination defects and promotes functional recovery in hypoxic brain injury. Brain 141, 85–98. 10.1093/brain/awx312 29244098 PMC6394402

[B29] Cullen C. L. Senesi M. Tang A. D. Clutterbuck M. T. Auderset L. O'Rourke M. E. (2019). Low-intensity transcranial magnetic stimulation promotes the survival and maturation of newborn oligodendrocytes in the adult mouse brain. Glia 67, 1462–1477. 10.1002/glia.23620 30989733 PMC6790715

[B30] Darmani G. Bergmann T. O. Butts Pauly K. Caskey C. F. de Lecea L. Fomenko A. (2022). Non-invasive transcranial ultrasound stimulation for neuromodulation. Clin. Neurophysiol. 135, 51–73. 10.1016/j.clinph.2021.12.010 35033772

[B31] de Angelis F. Bernardo A. Magnaghi V. Minghetti L. Tata A. M. (2012). Muscarinic receptor subtypes as potential targets to modulate oligodendrocyte progenitor survival, proliferation, and differentiation. Dev. Neurobiol. 72, 713–728. 10.1002/dneu.20976 21913336

[B32] Deisseroth K. (2015). Optogenetics: 10 years of microbial opsins in neuroscience. Nat. Neurosci. 18, 1213–1225. 10.1038/nn.4091 26308982 PMC4790845

[B33] Demerens C. Stankoff B. Logak M. Anglade P. Allinquant B. Couraud F. (1996). Induction of myelination in the central nervous system by electrical activity. Proc. Natl. Acad. Sci. U. S. A. 93, 9887–9892. 10.1073/pnas.93.18.9887 8790426 PMC38524

[B34] Deng S. Shu S. Zhai L. Xia S. Cao X. Li H. (2023). Optogenetic stimulation of mPFC alleviates white matter injury-related cognitive decline after chronic ischemia through adaptive myelination. Adv. Sci. (Weinh) 10, e2202976. 10.1002/advs.202202976 36529961 PMC9929132

[B35] Deshmukh V. A. Tardif V. Lyssiotis C. A. Green C. C. Kerman B. Kim H. J. (2013). A regenerative approach to the treatment of multiple sclerosis. Nature 502, 327–332. 10.1038/nature12647 24107995 PMC4431622

[B36] Dimitriadou V. Pang X. Theoharides T. C. (2000). Hydroxyzine inhibits experimental allergic encephalomyelitis (EAE) and associated brain mast cell activation. Int. J. Immunopharmacol. 22, 673–684. 10.1016/s0192-0561(00)00029-1 10884588

[B37] Dolgova N. Wei Z. Spink B. Gui L. Hua Q. Truong D. (2021). Low-field magnetic stimulation accelerates the differentiation of oligodendrocyte precursor cells via non-canonical TGF-beta signaling pathways. Mol. Neurobiol. 58, 855–866. 10.1007/s12035-020-02157-0 33037982

[B38] Domercq M. Perez-Samartin A. Aparicio D. Alberdi E. Pampliega O. Matute C. (2010). P2X7 receptors mediate ischemic damage to oligodendrocytes. Glia 58, 730–740. 10.1002/glia.20958 20029962

[B39] Doyle S. Hansen D. B. Vella J. Bond P. Harper G. Zammit C. (2018). Vesicular glutamate release from central axons contributes to myelin damage. Nat. Commun. 9, 1032. 10.1038/s41467-018-03427-1 29531223 PMC5847599

[B40] Etxeberria A. Mangin J. M. Aguirre A. Gallo V. (2010). Adult-born SVZ progenitors receive transient synapses during remyelination in corpus callosum. Nat. Neurosci. 13, 287–289. 10.1038/nn.2500 20173746 PMC4681435

[B41] Fannon J. Tarmier W. Fulton D. (2015). Neuronal activity and AMPA-type glutamate receptor activation regulates the morphological development of oligodendrocyte precursor cells. Glia 63, 1021–1035. 10.1002/glia.22799 25739948

[B42] Fields R. D. Dutta D. J. Belgrad J. Robnett M. (2017). Cholinergic signaling in myelination. Glia 65, 687–698. 10.1002/glia.23101 28101995

[B43] Fields R. D. (2008). White matter in learning, cognition and psychiatric disorders. Trends Neurosci. 31, 361–370. 10.1016/j.tins.2008.04.001 18538868 PMC2486416

[B44] Fulmer C. G. Vondran M. W. Stillman A. A. Huang Y. Hempstead B. L. Dreyfus C. F. (2014). Astrocyte-derived BDNF supports myelin protein synthesis after cuprizone-induced demyelination. J. Neurosci. 34, 8186–8196. 10.1523/JNEUROSCI.4267-13.2014 24920623 PMC4051974

[B45] Gautier H. O. Evans K. A. Volbracht K. James R. Sitnikov S. Lundgaard I. (2015). Neuronal activity regulates remyelination via glutamate signalling to oligodendrocyte progenitors. Nat. Commun. 6, 8518. 10.1038/ncomms9518 26439639 PMC4600759

[B46] Gibson E. M. Purger D. Mount C. W. Goldstein A. K. Lin G. L. Wood L. S. (2014). Neuronal activity promotes oligodendrogenesis and adaptive myelination in the mammalian brain. Science 344, 1252304. 10.1126/science.1252304 24727982 PMC4096908

[B47] Gonzalez-Fernandez E. Sanchez-Gomez M. V. Perez-Samartin A. Arellano R. O. Matute C. (2014). A3 Adenosine receptors mediate oligodendrocyte death and ischemic damage to optic nerve. Glia 62, 199–216. 10.1002/glia.22599 24311446

[B48] Green A. J. Gelfand J. M. Cree B. A. Bevan C. Boscardin W. J. Mei F. (2017). Clemastine fumarate as a remyelinating therapy for multiple sclerosis (ReBUILD): A randomised, controlled, double-blind, crossover trial. Lancet 390, 2481–2489. 10.1016/S0140-6736(17)32346-2 29029896

[B49] Hamilton N. B. Clarke L. E. Arancibia-Carcamo I. L. Kougioumtzidou E. Matthey M. Karadottir R. (2017). Endogenous GABA controls oligodendrocyte lineage cell number, myelination, and CNS internode length. Glia 65, 309–321. 10.1002/glia.23093 27796063 PMC5214060

[B50] Horner P. J. Gage F. H. (2000). Regenerating the damaged central nervous system. Nature 407, 963–970. 10.1038/35039559 11069169

[B51] Hu W. Chen Z. (2017). The roles of histamine and its receptor ligands in central nervous system disorders: An update. Pharmacol. Ther. 175, 116–132. 10.1016/j.pharmthera.2017.02.039 28223162

[B52] Huang J. X. Zhu B. L. Xu J. P. Zhou Z. Z. (2023). Advances in the development of phosphodiesterase 7 inhibitors. Eur. J. Med. Chem. 250, 115194. 10.1016/j.ejmech.2023.115194 36796299

[B53] Illes P. (2020). P2X7 receptors amplify CNS damage in neurodegenerative diseases. Int. J. Mol. Sci. 21, 5996. 10.3390/ijms21175996 32825423 PMC7504621

[B54] Imamura O. Arai M. Dateki M. Ogata T. Uchida R. Tomoda H. (2015). Nicotinic acetylcholine receptors mediate donepezil-induced oligodendrocyte differentiation. J. Neurochem. 135, 1086–1098. 10.1111/jnc.13294 26315944

[B55] Imamura O. Arai M. Dateki M. Oishi K. Takishima K. (2020). Donepezil-induced oligodendrocyte differentiation is mediated through estrogen receptors. J. Neurochem. 155, 494–507. 10.1111/jnc.14927 31778582

[B56] Imamura O. Arai M. Dateki M. Takishima K. (2017). Donepezil promotes differentiation of neural stem cells into mature oligodendrocytes at the expense of astrogenesis. J. Neurochem. 140, 231–244. 10.1111/jnc.13856 27664791

[B57] Imeri F. Stepanovska Tanturovska B. Zivkovic A. Enzmann G. Schwalm S. Pfeilschifter J. (2021). Novel compounds with dual S1P receptor agonist and histamine H(3) receptor antagonist activities act protective in a mouse model of multiple sclerosis. Neuropharmacology 186, 108464. 10.1016/j.neuropharm.2021.108464 33460688

[B58] Jensen S. K. Michaels N. J. Ilyntskyy S. Keough M. B. Kovalchuk O. Yong V. W. (2018). Multimodal enhancement of remyelination by exercise with a pivotal role for oligodendroglial PGC1α. Cell. Rep. 24, 3167–3179. 10.1016/j.celrep.2018.08.060 30232000

[B59] Jiang L. Cheng L. Chen H. Dai H. An D. Ma Q. (2021a). Histamine H2 receptor negatively regulates oligodendrocyte differentiation in neonatal hypoxic-ischemic white matter injury. J. Exp. Med. 218, e20191365. 10.1084/jem.20191365 32991666 PMC7527977

[B60] Jiang T. Luo J. Pan X. Zheng H. Yang H. Zhang L. (2021b). Physical exercise modulates the astrocytes polarization, promotes myelin debris clearance and remyelination in chronic cerebral hypoperfusion rats. Life Sci. 278, 119526. 10.1016/j.lfs.2021.119526 33894268

[B61] Kougioumtzidou E. Shimizu T. Hamilton N. B. Tohyama K. Sprengel R. Monyer H. (2017). Signalling through AMPA receptors on oligodendrocyte precursors promotes myelination by enhancing oligodendrocyte survival. Elife 6, e28080. 10.7554/eLife.28080 28608780 PMC5484614

[B62] Krasnow A. M. Attwell D. (2016). NMDA receptors: Power switches for oligodendrocytes. Neuron 91, 3–5. 10.1016/j.neuron.2016.06.023 27387644

[B63] Krasnow A. M. Ford M. C. Valdivia L. E. Wilson S. W. Attwell D. (2018). Regulation of developing myelin sheath elongation by oligodendrocyte calcium transients *in vivo* . Nat. Neurosci. 21, 24–28. 10.1038/s41593-017-0031-y 29230052 PMC6478117

[B64] Kukley M. Capetillo-Zarate E. Dietrich D. (2007). Vesicular glutamate release from axons in white matter. Nat. Neurosci. 10, 311–320. 10.1038/nn1850 17293860

[B65] Lai S. W. Chen J. H. Lin H. Y. Liu Y. S. Tsai C. F. Chang P. C. (2018). Regulatory effects of neuroinflammatory responses through brain-derived neurotrophic factor signaling in microglial cells. Mol. Neurobiol. 55, 7487–7499. 10.1007/s12035-018-0933-z 29427085

[B66] Larocca J. N. Almazan G. (1997). Acetylcholine agonists stimulate mitogen-activated protein kinase in oligodendrocyte progenitors by muscarinic receptors. J. Neurosci. Res. 50, 743–754. 10.1002/(SICI)1097-4547(19971201)50:5<743:AID-JNR11>3.0.CO;2-2 9418962

[B67] Larson V. A. Zhang Y. Bergles D. E. (2016). Electrophysiological properties of NG2(+) cells: Matching physiological studies with gene expression profiles. Brain Res. 1638, 138–160. 10.1016/j.brainres.2015.09.010 26385417 PMC4792778

[B68] Lee D. J. Lozano C. S. Dallapiazza R. F. Lozano A. M. (2019). Current and future directions of deep brain stimulation for neurological and psychiatric disorders. J. Neurosurg. 131, 333–342. 10.3171/2019.4.JNS181761 31370011

[B69] Li C. Xiao L. Liu X. Yang W. Shen W. Hu C. (2013). A functional role of NMDA receptor in regulating the differentiation of oligodendrocyte precursor cells and remyelination. Glia 61, 732–749. 10.1002/glia.22469 23440860

[B70] Li R. Zhang P. Zhang M. Yao Z. (2020). The roles of neuron-NG2 glia synapses in promoting oligodendrocyte development and remyelination. Cell. Tissue Res. 381, 43–53. 10.1007/s00441-020-03195-9 32236697

[B71] Li Z. He Y. Fan S. Sun B. (2015). Clemastine rescues behavioral changes and enhances remyelination in the cuprizone mouse model of demyelination. Neurosci. Bull. 31, 617–625. 10.1007/s12264-015-1555-3 26253956 PMC5563681

[B72] Liu H. Yang X. Yang J. Yuan Y. Wang Y. Zhang R. (2021). IL-17 inhibits oligodendrocyte progenitor cell proliferation and differentiation by increasing K(+) channel Kv1.3. Front. Cell. Neurosci. 15, 679413. 10.3389/fncel.2021.679413 34239419 PMC8258110

[B73] Liu J. Dupree J. L. Gacias M. Frawley R. Sikder T. Naik P. (2016). Clemastine enhances myelination in the prefrontal cortex and rescues behavioral changes in socially isolated mice. J. Neurosci. 36, 957–962. 10.1523/JNEUROSCI.3608-15.2016 26791223 PMC4719024

[B74] Liu Q. Li M. Whiteaker P. Shi F. D. Morley B. J. Lukas R. J. (2019). Attenuation in nicotinic acetylcholine receptor α9 and α10 subunit double knock-out mice of experimental autoimmune encephalomyelitis. Biomolecules 9, 827. 10.3390/biom9120827 31817275 PMC6995583

[B75] Loy B. D. Fling B. W. Sage K. M. Spain R. I. Horak F. B. (2019). Serum histidine is lower in fatigued women with multiple sclerosis. Fatigue 7, 69–80. 10.1080/21641846.2019.1611786 32440368 PMC7241417

[B76] Lubetzki C. Zalc B. Williams A. Stadelmann C. Stankoff B. (2020). Remyelination in multiple sclerosis: From basic science to clinical translation. Lancet Neurol. 19, 678–688. 10.1016/S1474-4422(20)30140-X 32702337

[B77] Lundgaard I. Luzhynskaya A. Stockley J. H. Wang Z. Evans K. A. Swire M. (2013). Neuregulin and BDNF induce a switch to NMDA receptor-dependent myelination by oligodendrocytes. PLoS Biol. 11, e1001743. 10.1371/journal.pbio.1001743 24391468 PMC3876980

[B78] Ma R. Z. Gao J. Meeker N. D. Fillmore P. D. Tung K. S. Watanabe T. (2002). Identification of Bps, an autoimmune disease locus, as histamine receptor H1. Science 297, 620–623. 10.1126/science.1072810 12142541

[B79] Maas D. A. Angulo M. C. (2021). Can enhancing neuronal activity improve myelin repair in multiple sclerosis? Front. Cell. Neurosci. 15, 645240. 10.3389/fncel.2021.645240 33708075 PMC7940692

[B80] Macrez R. Ortega M. C. Bardou I. Mehra A. Fournier A. van der Pol S. M. (2016). Neuroendothelial NMDA receptors as therapeutic targets in experimental autoimmune encephalomyelitis. Brain 139, 2406–2419. 10.1093/brain/aww172 27435092

[B81] Makowiecki K. Stevens N. Cullen C. L. Zarghami A. Nguyen P. T. Johnson L. (2022). Safety of low-intensity repetitive transcranial magneTic brAin stimUlation foR people living with mUltiple sclerosis (TAURUS): Study protocol for a randomised controlled trial. Trials 23, 626. 10.1186/s13063-022-06526-z 35922816 PMC9347125

[B82] Maldonado P. P. Velez-Fort M. Levavasseur F. Angulo M. C. (2013). Oligodendrocyte precursor cells are accurate sensors of local K+ in mature gray matter. J. Neurosci. 33, 2432–2442. 10.1523/JNEUROSCI.1961-12.2013 23392672 PMC6619152

[B83] Mandolesi G. Bullitta S. Fresegna D. de Vito F. Rizzo F. R. Musella A. (2019). Voluntary running wheel attenuates motor deterioration and brain damage in cuprizone-induced demyelination. Neurobiol. Dis. 129, 102–117. 10.1016/j.nbd.2019.05.010 31100354

[B84] Martin-Alvarez R. Paul-Fernandez N. Palomo V. Gil C. Martinez A. Mengod G. (2017). A preliminary investigation of phoshodiesterase 7 inhibitor VP3.15 as therapeutic agent for the treatment of experimental autoimmune encephalomyelitis mice. J. Chem. Neuroanat. 80, 27–36. 10.1016/j.jchemneu.2016.12.001 28007551

[B85] Mckenzie I. A. Ohayon D. Li H. de Faria J. P. Emery B. Tohyama K. (2014). Motor skill learning requires active central myelination. Science 346, 318–322. 10.1126/science.1254960 25324381 PMC6324726

[B86] Medina-Rodriguez E. M. Arenzana F. J. Pastor J. Redondo M. Palomo V. Garcia de Sola R. (2013). Inhibition of endogenous phosphodiesterase 7 promotes oligodendrocyte precursor differentiation and survival. Cell. Mol. Life Sci. 70, 3449–3462. 10.1007/s00018-013-1340-2 23661015 PMC11113628

[B87] Medina-Rodriguez E. M. Bribian A. Boyd A. Palomo V. Pastor J. Lagares A. (2017). Promoting *in vivo* remyelination with small molecules: A neuroreparative pharmacological treatment for multiple sclerosis. Sci. Rep. 7, 43545. 10.1038/srep43545 28256546 PMC5335257

[B88] Mei F. Lehmann-Horn K. Shen Y. A. Rankin K. A. Stebbins K. J. Lorrain D. S. (2016). Accelerated remyelination during inflammatory demyelination prevents axonal loss and improves functional recovery. Elife 5, e18246. 10.7554/eLife.18246 27671734 PMC5039026

[B89] Mensch S. Baraban M. Almeida R. Czopka T. Ausborn J. el Manira A. (2015). Synaptic vesicle release regulates myelin sheath number of individual oligodendrocytes *in vivo* . Nat. Neurosci. 18, 628–630. 10.1038/nn.3991 25849985 PMC4427868

[B90] Micu I. Jiang Q. Coderre E. Ridsdale A. Zhang L. Woulfe J. (2006). NMDA receptors mediate calcium accumulation in myelin during chemical ischaemia. Nature 439, 988–992. 10.1038/nature04474 16372019

[B91] Mitew S. Gobius I. Fenlon L. R. Mcdougall S. J. Hawkes D. Xing Y. L. (2018). Pharmacogenetic stimulation of neuronal activity increases myelination in an axon-specific manner. Nat. Commun. 9, 306. 10.1038/s41467-017-02719-2 29358753 PMC5778130

[B92] Mooshekhian A. Sandini T. Wei Z. van Bruggen R. Li H. Li X. M. (2022). Low-field magnetic stimulation improved cuprizone-induced depression-like symptoms and demyelination in female mice. Exp. Ther. Med. 23, 210. 10.3892/etm.2022.11133 35126713 PMC8796645

[B93] Musio S. Gallo B. Scabeni S. Lapilla M. Poliani P. L. Matarese G. (2006). A key regulatory role for histamine in experimental autoimmune encephalomyelitis: Disease exacerbation in histidine decarboxylase-deficient mice. J. Immunol. 176, 17–26. 10.4049/jimmunol.176.1.17 16365391

[B94] Nagy B. Hovhannisyan A. Barzan R. Chen T. J. Kukley M. (2017). Different patterns of neuronal activity trigger distinct responses of oligodendrocyte precursor cells in the corpus callosum. PLoS Biol. 15, e2001993. 10.1371/journal.pbio.2001993 28829781 PMC5567905

[B95] Nicholson M. Wood R. J. Gonsalvez D. G. Hannan A. J. Fletcher J. L. Xiao J. (2022). Remodelling of myelinated axons and oligodendrocyte differentiation is stimulated by environmental enrichment in the young adult brain. Eur. J. Neurosci. 56, 6099–6114. 10.1111/ejn.15840 36217300 PMC10092722

[B96] Nociti V. Romozzi M. (2023). The role of BDNF in multiple sclerosis neuroinflammation. Int. J. Mol. Sci. 24, 8447. 10.3390/ijms24098447 37176155 PMC10178984

[B97] Oliveria S. F. Rodriguez R. L. Bowers D. Kantor D. Hilliard J. D. Monari E. H. (2017). Safety and efficacy of dual-lead thalamic deep brain stimulation for patients with treatment-refractory multiple sclerosis tremor: A single-centre, randomised, single-blind, pilot trial. Lancet Neurol. 16, 691–700. 10.1016/S1474-4422(17)30166-7 28642125

[B98] Ortiz F. C. Habermacher C. Graciarena M. Houry P. Y. Nishiyama A. Nait Oumesmar B. (2019). Neuronal activity *in vivo* enhances functional myelin repair. JCI Insight 5, e123434. 10.1172/jci.insight.123434 30896448 PMC6538342

[B99] Oyanguren-Desez O. Rodriguez-Antiguedad A. Villoslada P. Domercq M. Alberdi E. Matute C. (2011). Gain-of-function of P2X7 receptor gene variants in multiple sclerosis. Cell. Calcium 50, 468–472. 10.1016/j.ceca.2011.08.002 21906809

[B100] Paez P. M. Fulton D. Colwell C. S. Campagnoni A. T. (2009). Voltage-operated Ca(2+) and Na(+) channels in the oligodendrocyte lineage. J. Neurosci. Res. 87, 3259–3266. 10.1002/jnr.21938 19021296

[B101] Paez P. M. Lyons D. A. (2020). Calcium signaling in the oligodendrocyte lineage: Regulators and consequences. Annu. Rev. Neurosci. 43, 163–186. 10.1146/annurev-neuro-100719-093305 32075518

[B102] Palma A. Chara J. C. Montilla A. Otxoa-de-Amezaga A. Ruiz-Jaen F. Planas A. M. (2022). Clemastine induces an impairment in developmental myelination. Front. Cell. Dev. Biol. 10, 841548. 10.3389/fcell.2022.841548 35372341 PMC8970281

[B103] Pan S. Chan J. R. (2021). Clinical applications of myelin plasticity for remyelinating therapies in multiple sclerosis. Ann. Neurol. 90, 558–567. 10.1002/ana.26196 34402546 PMC8555870

[B104] Panula P. Nuutinen S. (2013). The histaminergic network in the brain: Basic organization and role in disease. Nat. Rev. Neurosci. 14, 472–487. 10.1038/nrn3526 23783198

[B105] Passani M. B. Ballerini C. (2012). Histamine and neuroinflammation: Insights from murine experimental autoimmune encephalomyelitis. Front. Syst. Neurosci. 6, 32. 10.3389/fnsys.2012.00032 22563309 PMC3342557

[B106] Passani M. B. Blandina P. (2011). Histamine receptors in the CNS as targets for therapeutic intervention. Trends Pharmacol. Sci. 32, 242–249. 10.1016/j.tips.2011.01.003 21324537

[B107] Piovesana R. Reid A. J. Tata A. M. (2022). Emerging roles of cholinergic receptors in schwann cell development and plasticity. Biomedicines 11, 41. 10.3390/biomedicines11010041 36672549 PMC9855772

[B108] Pudasaini S. Friedrich V. Buhrer C. Endesfelder S. Scheuer T. Schmitz T. (2022). Postnatal myelination of the immature rat cingulum is regulated by GABA(B) receptor activity. Dev. Neurobiol. 82, 16–28. 10.1002/dneu.22853 34605209

[B109] Rafiee Zadeh A. Falahatian M. Alsahebfosoul F. (2018). Serum levels of histamine and diamine oxidase in multiple sclerosis. Am. J. Clin. Exp. Immunol. 7, 100–105. 30697467 PMC6334194

[B110] Ragheb F. Molina-Holgado E. Cui Q. L. Khorchid A. Liu H. N. Larocca J. N. (2001). Pharmacological and functional characterization of muscarinic receptor subtypes in developing oligodendrocytes. J. Neurochem. 77, 1396–1406. 10.1046/j.1471-4159.2001.00356.x 11389190

[B111] Rivera A. D. Chacon-de-la-Rocha I. Pieropan F. Papanikolau M. Azim K. Butt A. M. (2021). Keeping the ageing brain wired: A role for purine signalling in regulating cellular metabolism in oligodendrocyte progenitors. Pflugers Arch. 473, 775–783. 10.1007/s00424-021-02544-z 33712969 PMC8076121

[B112] Rivera A. Vanzulli I. Butt A. M. (2016). A central role for ATP signalling in glial interactions in the CNS. Curr. Drug Targets 17, 1829–1833. 10.2174/1389450117666160711154529 27400972

[B113] Roth B. L. (2016). DREADDs for neuroscientists. Neuron 89, 683–694. 10.1016/j.neuron.2016.01.040 26889809 PMC4759656

[B114] Sahel A. Ortiz F. C. Kerninon C. Maldonado P. P. Angulo M. C. Nait-Oumesmar B. (2015). Alteration of synaptic connectivity of oligodendrocyte precursor cells following demyelination. Front. Cell. Neurosci. 9, 77. 10.3389/fncel.2015.00077 25852473 PMC4362325

[B115] Saligrama N. Case L. K. Del Rio R. Noubade R. Teuscher C. (2013). Systemic lack of canonical histamine receptor signaling results in increased resistance to autoimmune encephalomyelitis. J. Immunol. 191, 614–622. 10.4049/jimmunol.1203137 23772030 PMC3747232

[B116] Saligrama N. Case L. K. Krementsov D. N. Teuscher C. (2014). Histamine H₂ receptor signaling × environment interactions determine susceptibility to experimental allergic encephalomyelitis. FASEB J. 28, 1898–1909. 10.1096/fj.13-239939 24371118 PMC3963021

[B117] Salter M. G. Fern R. (2005). NMDA receptors are expressed in developing oligodendrocyte processes and mediate injury. Nature 438, 1167–1171. 10.1038/nature04301 16372012

[B118] Santiago Gonzalez D. A. Cheli V. T. Zamora N. N. Lama T. N. Spreuer V. Murphy G. G. (2017). Conditional deletion of the L-type calcium channel Cav1.2 in NG2-positive cells impairs remyelination in mice. J. Neurosci. 37, 10038–10051. 10.1523/JNEUROSCI.1787-17.2017 28899915 PMC5647766

[B119] Schwartzbach C. J. Grove R. A. Brown R. Tompson D. Then Bergh F. Arnold D. L. (2017). Lesion remyelinating activity of GSK239512 versus placebo in patients with relapsing-remitting multiple sclerosis: A randomised, single-blind, phase II study. J. Neurol. 264, 304–315. 10.1007/s00415-016-8341-7 27888416 PMC5306088

[B120] Serrano-Regal M. P. Bayon-Cordero L. Chara Ventura J. C. Ochoa-Bueno B. I. Tepavcevic V. Matute C. (2022). GABA(B) receptor agonist baclofen promotes central nervous system remyelination. Glia 70, 2426–2440. 10.1002/glia.24262 35980256 PMC9804779

[B121] Shi Y. Li Z. Chen R. Zhang J. Hu X. He C. (2017). Immethridine, histamine H(3)-receptor (H(3)R) agonist, alleviated experimental autoimmune encephalomyelitis via inhibiting the function of dendritic cells. Oncotarget 8, 75038–75049. 10.18632/oncotarget.20500 29088843 PMC5650398

[B122] Smith K. J. Blakemore W. F. Mcdonald W. I. (1979). Central remyelination restores secure conduction. Nature 280, 395–396. 10.1038/280395a0 460414

[B123] Song F. Hong X. Cao J. Ma G. Han Y. Cepeda C. (2018). Kir4.1 channels in NG2-glia play a role in development, potassium signaling, and ischemia-related myelin loss. Commun. Biol. 1, 80. 10.1038/s42003-018-0083-x 30271961 PMC6123808

[B124] Spitzer S. Volbracht K. Lundgaard I. Karadottir R. T. (2016). Glutamate signalling: A multifaceted modulator of oligodendrocyte lineage cells in health and disease. Neuropharmacology 110, 574–585. 10.1016/j.neuropharm.2016.06.014 27346208

[B125] Stevens B. Porta S. Haak L. L. Gallo V. Fields R. D. (2002). Adenosine: A neuron-glial transmitter promoting myelination in the CNS in response to action potentials. Neuron 36, 855–868. 10.1016/s0896-6273(02)01067-x 12467589 PMC1201407

[B126] Sun W. Matthews E. A. Nicolas V. Schoch S. Dietrich D. (2016). NG2 glial cells integrate synaptic input in global and dendritic calcium signals. Elife 5, e16262. 10.7554/eLife.16262 27644104 PMC5052029

[B127] Takeda M. Nelson D. J. Soliven B. (1995). Calcium signaling in cultured rat oligodendrocytes. Glia 14, 225–236. 10.1002/glia.440140308 7591034

[B128] Teuscher C. Subramanian M. Noubade R. Gao J. F. Offner H. Zachary J. F. (2007). Central histamine H3 receptor signaling negatively regulates susceptibility to autoimmune inflammatory disease of the CNS. Proc. Natl. Acad. Sci. U. S. A. 104, 10146–10151. 10.1073/pnas.0702291104 17548817 PMC1891222

[B129] Thornton M. A. Hughes E. G. (2020). Neuron-oligodendroglia interactions: Activity-dependent regulation of cellular signaling. Neurosci. Lett. 727, 134916. 10.1016/j.neulet.2020.134916 32194135 PMC7295072

[B130] Tong L. Y. Deng Y. B. du W. H. Zhou W. Z. Liao X. Y. Jiang X. (2022). Clemastine promotes differentiation of oligodendrocyte progenitor cells through the activation of ERK1/2 via muscarinic receptors after spinal cord injury. Front. Pharmacol. 13, 914153. 10.3389/fphar.2022.914153 35865954 PMC9294397

[B131] Tong X. P. Li X. Y. Zhou B. Shen W. Zhang Z. J. Xu T. L. (2009). Ca(2+) signaling evoked by activation of Na(+) channels and Na(+)/Ca(2+) exchangers is required for GABA-induced NG2 cell migration. J. Cell. Biol. 186, 113–128. 10.1083/jcb.200811071 19596850 PMC2712990

[B132] Tsiperson V. Huang Y. Bagayogo I. Song Y. Vondran M. W. Dicicco-Bloom E. (2015). Brain-derived neurotrophic factor deficiency restricts proliferation of oligodendrocyte progenitors following cuprizone-induced demyelination. ASN Neuro 7, 1759091414566878. 10.1177/1759091414566878 25586993 PMC4720179

[B133] Tsutsui S. Schnermann J. Noorbakhsh F. Henry S. Yong V. W. Winston B. W. (2004). A1 adenosine receptor upregulation and activation attenuates neuroinflammation and demyelination in a model of multiple sclerosis. J. Neurosci. 24, 1521–1529. 10.1523/JNEUROSCI.4271-03.2004 14960625 PMC6730323

[B134] Velez-Fort M. Audinat E. Angulo M. C. (2009). Functional alpha 7-containing nicotinic receptors of NG2-expressing cells in the hippocampus. Glia 57, 1104–1114. 10.1002/glia.20834 19170184

[B135] Volonte C. Apolloni S. Amadio S. (2022). The histamine and multiple sclerosis alliance: Pleiotropic actions and functional validation. Curr. Top. Behav. Neurosci. 59, 217–239. 10.1007/7854_2021_240 34432258

[B136] Vondran M. W. Clinton-Luke P. Honeywell J. Z. Dreyfus C. F. (2010). BDNF+/- mice exhibit deficits in oligodendrocyte lineage cells of the basal forebrain. Glia 58, 848–856. 10.1002/glia.20969 20091777 PMC2851835

[B137] Vondran M. W. Singh H. Honeywell J. Z. Dreyfus C. F. (2011). Levels of BDNF impact oligodendrocyte lineage cells following a cuprizone lesion. J. Neurosci. 31, 14182–14190. 10.1523/JNEUROSCI.6595-10.2011 21976503 PMC3203635

[B138] Wang Z. Baharani A. Wei Z. Truong D. Bi X. Wang F. (2021). Low field magnetic stimulation promotes myelin repair and cognitive recovery in chronic cuprizone mouse model. Clin. Exp. Pharmacol. Physiol. 48, 1090–1102. 10.1111/1440-1681.13490 33638234

[B139] Welsh T. G. Kucenas S. (2018). Purinergic signaling in oligodendrocyte development and function. J. Neurochem. 145, 6–18. 10.1111/jnc.14315 29377124 PMC5937939

[B140] Wessler I. Kirkpatrick C. J. Racke K. (1999). The cholinergic 'pitfall': Acetylcholine, a universal cell molecule in biological systems, including humans. Clin. Exp. Pharmacol. Physiol. 26, 198–205. 10.1046/j.1440-1681.1999.03016.x 10081614

[B141] Wilson D. M. Apps J. Bailey N. Bamford M. J. Beresford I. J. Brackenborough K. (2013). Identification of clinical candidates from the benzazepine class of histamine H3 receptor antagonists. Bioorg Med. Chem. Lett. 23, 6890–6896. 10.1016/j.bmcl.2013.09.090 24269482

[B142] Wooliscroft L. Silbermann E. Cameron M. Bourdette D. (2019). Approaches to remyelination therapies in multiple sclerosis. Curr. Treat. Options Neurol. 21, 34. 10.1007/s11940-019-0574-1 31250211

[B143] Wrann C. D. White J. P. Salogiannnis J. Laznik-Bogoslavski D. Wu J. Ma D. (2013). Exercise induces hippocampal BDNF through a PGC-1α/FNDC5 pathway. Cell. Metab. 18, 649–659. 10.1016/j.cmet.2013.09.008 24120943 PMC3980968

[B144] Xiao L. Ohayon D. Mckenzie I. A. Sinclair-Wilson A. Wright J. L. Fudge A. D. (2016). Rapid production of new oligodendrocytes is required in the earliest stages of motor-skill learning. Nat. Neurosci. 19, 1210–1217. 10.1038/nn.4351 27455109 PMC5008443

[B145] Yang F. Y. Huang L. H. Wu M. T. Pan Z. Y. (2022). Ultrasound neuromodulation reduces demyelination in a rat model of multiple sclerosis. Int. J. Mol. Sci. 23, 10034. 10.3390/ijms231710034 36077437 PMC9456451

[B146] Yao S. Q. Li Z. Z. Huang Q. Y. Li F. Wang Z. W. Augusto E. (2012). Genetic inactivation of the adenosine A(2A) receptor exacerbates brain damage in mice with experimental autoimmune encephalomyelitis. J. Neurochem. 123, 100–112. 10.1111/j.1471-4159.2012.07807.x 22639925

[B147] Yoon H. Kleven A. Paulsen A. Kleppe L. Wu J. Ying Z. (2016). Interplay between exercise and dietary fat modulates myelinogenesis in the central nervous system. Biochim. Biophys. Acta 1862, 545–555. 10.1016/j.bbadis.2016.01.019 26826016 PMC4788558

[B148] Zhao X. Li Y. Tian Q. Zhu B. Zhao Z. (2019). Repetitive transcranial magnetic stimulation increases serum brain-derived neurotrophic factor and decreases interleukin-1β and tumor necrosis factor-α in elderly patients with refractory depression. J. Int. Med. Res. 47, 1848–1855. 10.1177/0300060518817417 30616482 PMC6567781

[B149] Zhou Y. An D. Xu Y. Zhou Y. Li Q. Dai H. (2022). Activation of glutamatergic neurons in the somatosensory cortex promotes remyelination in ischemic vascular dementia. Fundam. Res. 10.1016/j.fmre.2022.08.007 PMC11197523 38933843

[B150] Zhou Y. Zhang J. Wang L. Chen Y. Wan Y. He Y. (2017). Interleukin-1β impedes oligodendrocyte progenitor cell recruitment and white matter repair following chronic cerebral hypoperfusion. Brain Behav. Immun. 60, 93–105. 10.1016/j.bbi.2016.09.024 27663285

[B151] Zonouzi M. Scafidi J. Li P. Mcellin B. Edwards J. Dupree J. L. (2015). GABAergic regulation of cerebellar NG2 cell development is altered in perinatal white matter injury. Nat. Neurosci. 18, 674–682. 10.1038/nn.3990 25821912 PMC4459267

[B152] Zorn A. Baillie G. (2023). Phosphodiesterase 7 as a therapeutic target - where are we now? Cell. Signal 108, 110689. 10.1016/j.cellsig.2023.110689 37120115

